# Unveiling Urinary Mutagenicity by the Ames Test for Occupational Risk Assessment: A Systematic Review

**DOI:** 10.3390/ijerph192013074

**Published:** 2022-10-11

**Authors:** Bela Barros, Marta Oliveira, Simone Morais

**Affiliations:** REQUIMTE-LAQV, Instituto Superior de Engenharia do Porto, Instituto Politécnico do Porto, Rua Dr. António Bernardino de Almeida 431, 4249-015 Porto, Portugal

**Keywords:** urinary biomarker, toxic exposure, occupational health, mutagenicity

## Abstract

Occupational exposure may involve a variety of toxic compounds. A mutagenicity analysis using the Ames test can provide valuable information regarding the toxicity of absorbed xenobiotics. Through a search of relevant databases, this systematic review gathers and critically discusses the published papers (excluding other types of publications) from 2001–2021 that have assessed urinary mutagenicity (Ames test with *Salmonella typhimurium*) in an occupational exposure context. Due to the heterogeneity of the study methods, a meta-analysis could not be conducted. The characterized occupations were firefighters, traffic policemen, bus drivers, mail carriers, coke oven and charcoal workers, chemical laboratory staff, farmers, pharmacy workers, and professionals from several other industrial sectors. The genetically modified bacterial strains (histidine dependent) TA98, TA100, YG1041, YG1021, YG1024 and YG1042 have been used for the health risk assessment of individual (e.g., polycyclic aromatic hydrocarbons) and mixtures of compounds (e.g., diesel engine exhaust, fire smoke, industrial fumes/dyes) in different contexts. Although comparison of the data between studies is challenging, urinary mutagenicity can be very informative of possible associations between work-related exposure and the respective mutagenic potential. Careful interpretation of results and their direct use for occupational health risk assessment are crucial and yet complex; the use of several strains is highly recommended since individual and/or synergistic effects of complex exposure to xenobiotics can be overlooked. Future studies should improve the methods used to reach a standardized protocol for specific occupational environments to strengthen the applicability of the urinary mutagenicity assay and reduce inter- and intra-individual variability and exposure source confounders.

## 1. Introduction

Nowadays, our surrounding environment can be characterized by a complexity of natural and man-made xenobiotics. Human exposure to environment contaminants (by ingestion, inhalation, dermal/eye contact) can have serious consequences for health [1]. The absorbed dose and the biochemical properties (persistence, toxicity and latency, among others) determine the mode of action of a given xenobiotic in the body and which physiological mechanisms and macromolecules (e.g., DNA) are affected [1]. Some occupations such as firefighting, charcoal, coke oven, and industrial workers can be exposed to a variety of toxic chemicals during their routine activities [2,3]. For instance, the breathable air may contain chemicals from among 500 genotoxic chemicals [4]. Such chemicals can be released from industrial manufacturing, fire emissions and fuel combustion [5] with some of them being classified as known carcinogens to humans [6]. Nevertheless, polycyclic aromatic hydrocarbons (PAHs) and particulate matter (PM) are the most widely studied pollutants, especially fine (PM with an aerodynamic diameter less than 2.5 µm, i.e., PM_2.5_) and ultrafine (PM of size less than 0.1 µm, i.e., PM_0.1_) particles, which can cross the pulmonary epithelium, reach the blood stream and cause damage to internal organs [7]. Such exposures can be responsible for the development of respiratory and cardiovascular diseases [6,8,9]. Additionally, given the mutagenic capacity of some chemicals, long term exposure can result in cancer development. There are several pathways to disease development, which can be monitored by biological sample analysis (e.g., urine, blood, exhaled breath) [10]. Biomonitoring of chemicals and their effects can have a significant impact on the detection and mitigation of health consequences among the general and occupationally exposed population. Over almost 50 years, the *Salmonella* (Ames) mutagenicity assay has been used in human biomonitoring to evaluate genotoxic exposure, regardless the route of exposure [11]. This mutagenicity test is based on the *Salmonella*/microsome test, which since its introduction in 1973, has been widely used as biological assay to assess the mutagenic potential of individual compounds or mixtures that may endanger human health [11,12,13]. Moreover, because biomarker analysis is directly linked with the internal dose burden, biomonitoring can be a less biased surrogate for exposure assessment when compared with environmental monitoring (based on air, soil or water) [14]. Consequently, this urinary toxicological assay can be applied in an occupational context to help characterize the health risks associated with a given work-related activity. This systematic review aims to gather information published over the last 20 years on the application of urinary mutagenicity (UM) testing in occupational biomonitoring. UM methods and reported levels are compared and discussed in relation to occupational risk assessment.

## 2. Materials and Methods

**Figure 1:**
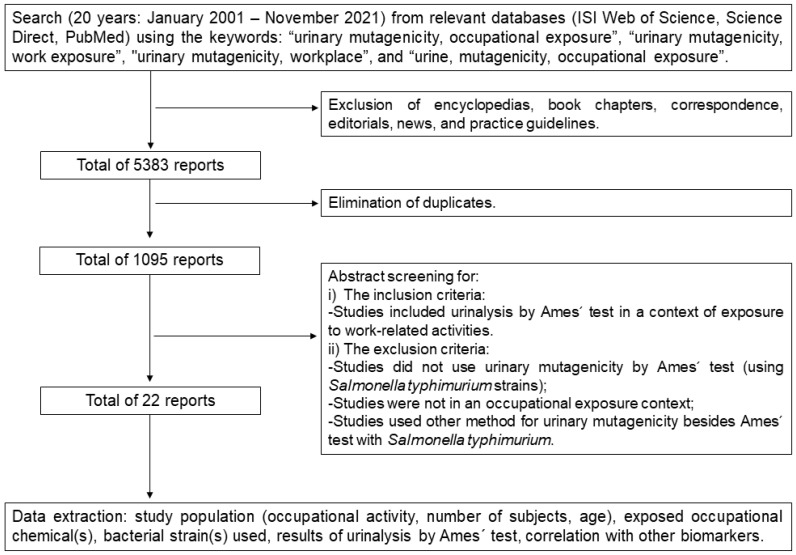
Flow chart showing the systematic methodology, eligibility criteria, and criteria for data extraction from the selected studies.

Figure 1 shows the steps taken to conduct this systematic review. Several databases (ISI Web of Science, Science Direct, and PubMed) were used to search for published articles between January 2001 and November 2021. The keywords used for the search on the databases were as follows: “urinary mutagenicity, occupational exposure”, “urinary mutagenicity, work exposure”, “urinary mutagenicity, workplace”, and “urine, mutagenicity, occupational exposure”. No further search strings were included. Encyclopedia entries, book chapters, correspondence, editorials, news, and practice guidelines were excluded. A total of 5383 results were found. By elimination of duplicates and abstract screening, 22 studies were selected. Studies that did not report results of UM by the Ames test using *Salmonella typhimurium* in association with occupational-related exposure were excluded. No automation tools were used for data synthesis and analysis, no assumptions were made about any missing data. The following data were collected from each study: study population (occupational activity, number of subjects, age), exposed occupational chemical(s), bacterial strain(s) used, UM results, correlation with other biomarkers. To evaluate the quality of the data extraction and assessment of the methodological (and risk of bias) quality of the selected studies based on the defined eligibility criteria, information retrieved from each study was checked twice by all authors, and the data related with potential confounder variables including the number of participating subjects, individual confounders (e.g., age, sex, tobacco consumption, etc.), strains used, use of positive and negative controls in the study, and the presentation of results with creatinine-adjustment were considered. Due to the heterogeneity of methods and differences in result units reported among the selected studies, a meta-analysis could not be conducted.

## 3. The Urinary Mutagenicity Method

The Ames bioassay, a mutagenicity test performed with the enterobacterium *Salmonella typhimurium*, was developed in the 1970s. Since then, several *S. typhimurium* strains have been designed for different sample analyses depending on the chosen application [15]. The methodological steps for evaluating the mutagenic potential of the urine test sample include the exposure of genetically modified histidine-dependent microorganisms to different concentrations of the test sample in a histidine-free medium [16]. Base substitutions or frameshift mutations within the inactivated histidine synthesis gene are responsible for the activation of histidine production, which causes the cells to reproduce. Therefore, upon exposure, the grown mutated colonies (i.e., revertants) can be observed and counted [15,16,17]. Urine sterilization and pre-treatment are also required to prevent the growth of other colonies besides the test trains. Moreover, since some pollutants can be excreted in their unmetabolized form, the bacterial strains must be metabolically active to mimic human metabolism. The bioactivation of these compounds can be provided by endogenous bacterial enzymes or an exogenous system containing cytochrome P450 enzymes from rat (e.g., mammalian liver S9, i.e., S9 mix), fish or human liver homogenates and cofactors [2,15]. Depending on the choice (with or without metabolism activation), there are guidelines with reference chemicals that can be used as positive controls of mutagenicity (Table 1). Note that the choice of controls is also dependent on the selected strain(s) and their characteristic number of spontaneous mutations [16].

### UM Limitations

The overall mutagenicity of mixtures, accounting for their interactions with other compounds, is more similar to what occurs in a living organism. Therefore, in addition to using bacteria (which have less demanding cell culture and maintenance in comparison with eukaryotic cell culture), the ability to analyze a mixture of chemicals, instead of analyzing each individually, is the main advantage of this bioassay. However, the UM Ames test does not account for the internal biological interactions before the excretion of the chemicals that can occur in a human body, i.e., some individuals can be more susceptible to mutagenic agents than others, or individual metabolic characteristics can influence the overall magnitude of effects that the person can have. Therefore, a positive UM result does not necessarily mean an ongoing disease development. Another limitation of the *Salmonella*/microsome assay is the misinterpretation of results when using only a few or a single strain, which can mask the overall mutagenic effect. If one of the bacteria in the mixture is not sensitive to the presence of the chemical, there could be a less positive or even negative result, which can be misinterpreted as the amount of exposure (potentially less) rather than the poorer choice of the strains used.

## 4. Test Strains Used for Occupational Biomonitoring

Over the last 20 years, distinct strains have been used for UM assessment in occupational biomonitoring studies (Table 2). TA98 and TA100 were the first to be developed, followed by the improved test strains YG1041, YG1021 and YG1024 derived from the parental strain TA98 and YG1042, resulting from TA100 [18,19,20].

Strain TA98 has a mutation on the *hisD3052* gene, in the histidine operon, which prevents the expression of histidinol dehydrogenase enzyme (histidine synthesis) [15,21]. In this way, mutagens that promote DNA deletions or insertions shifting the way the DNA sequence is read (frameshift mutations) can restore DNA expression of histidinol dehydrogenase, thus activating histidine synthesis in TA98 strains. In addition to the *hisD3052* mutation, TA98 has a *rfa* mutation (partial loss of lipopolysaccharides that compose the bacterial wall) designed to allow a higher permeability to larger molecules that, otherwise, could not enter the cell [15]. To increase the sensibility of this strain, the plasmid *pKM101*, which transports the *mucAB* gene (error-prone DNA repair system), increases the spontaneous and induced mutagenicity of the TA98 strain [22]. Lastly, TA98 has a *uvrB* deletion that prevents the expression of the error-free DNA excision repair system. For technical reasons, this deletion also prevents biotin synthesis; thus, TA98’s medium needs to supply biotin for bacterial growth [15].

On the other hand, strain TA100 can be used to detect base-pair substitutions in a suppressed gene that codifies the first enzyme responsible for histidine biosynthesis (*hisG46* mutation) [15]. This base-pair substitution results in an amino acid change in the protein, i.e., proline is replaced by leucine. Therefore, mutagens that reverse the *hisG46* mutation allow histidine synthesis to proceed and promote bacterial growth in a histidine-free medium. Maron and Ames (1983) developed the improved TA97, a strain in which the *hisO1242* mutation causes a constitutive expression of the histidine operon and produces a slight enhancement of frameshift (*hisD6610*) mutations [15]. Furthermore, TA102 is a strain carrying an ochre mutation on a multicopy plasmid, *hisG428* (*pAQ 1*), which instead of the *urvB* mutation, has the *hisG428* mutation and a tetracycline resistance gene [15]. This strain allows the detection of mutagenicity caused by other less characterized agents, i.e., formaldehyde, X-rays, glyoxal, phenylhydrazine, ultraviolet light, streptonigrin, psoralens bleomycin, mitomycin C and various hydroperoxides [15]. On the other hand, Hagiwara et al. (1993) developed the YG1041 and YG1042 strains from the TA98 and TA100 strains, respectively, in which the authors added the *pYG233* plasmid. Besides the parental strain, the difference between the two is that instead of kanamycin resistance (YG1041), YG1042 strain presents ampicillin resistance [23]. Regarding YG1041, the presence of this plasmid increases the levels of nitroreductases and O-acetyltransferases, which are more sensitive to nitroarenes and aromatic amines exposure [23]. Nitro-PAHs can covalently bind to DNA and originate DNA-adducts, which in turn are responsible for the frameshift mutations that were already detectable by the TA98 strain. Therefore, the YG1041 strain has an increased sensitivity to compounds of the nitro-, hydroxylamino- and amino-aromatic groups that surpasses its parental strain (TA98) [18,23,24,25,26].

Watanabe et al. (1990) developed the strain YG1024 that is also derived from TA98 after the addition of the *pBR322* plasmid harboring the acetyltransferase gene of the *S. typhimurium* TA1538 strain. This improved strain has a higher N-hydroxyarylanine O-acetyltransferase activity that displays an increased susceptibility to mutagens such as aromatic amines and nitroarenes [19,27]. The applicability of this strain to the complex mixture of chemicals in the urine of smokers has also been demonstrated [20,28]. Alternatively, the strand YG1021 presents a higher level of nitroreductases and has demonstrated a higher efficiency in the detection of a variety of mutagenic nitro compounds [29]. This characteristic is given by the addition of a plasmid containing the nitroreductase gene from *S. typhimurium* TA1538 into the strain TA98 [29]. To test the efficiency of YG1021 to detect mutagenic activity, a test was performed with exposure to three diphenyl ether pesticides and the attained results showed a 10-fold higher nitroreductase activity than with the parental strain TA98 [29]. In addition, Kuenemann-Migeot et al. (1997) compared all three strains (TA98, YG1024 and YG1021) for UM analysis of smokers, demonstrating that for this application: “YG1024 was more sensitive in detecting pro- mutagenicity in smokers’ urine compared to the conventional strain TA98, while the strain YG1021 did not show any increased sensitivity.” In fact, YG1024 is more sensitive to 2-aminofluorene and 2-aminoanthrance (the main aromatic amine in cigarette smoke) than its parental strain TA98 (it is more responsive to nitro-amines) [20,30]. An interesting finding from these previous studies was that glucurono- and sulfo-conjugated compounds only became mutagenic after metabolic activation, which means that the hydrolysis of urinary extracts decreases the mutagenic activity of these compounds in TA98 strains. However, in YG1024, the pre-treatment of urine extracts with *β*-glucuronidase releases aromatic aminated derivatives that are more mutagenic and less cytotoxic to this specific strain. On the other hand, hydrolysis with arylsulfatase increases the mutagenicity of urine extracts in both strains (TA98 and YG1024). The use of both YG1021 and YG1024 allowed the detection of amino aromatic pro-mutagens existing in the urinary extracts of smokers mainly as glucurono- and sulfo-conjugated compounds [20]. However, no information could be obtained regarding specific chemicals such as nitroamines or PAHs, which are known constituents of tobacco smoke.

## 5. Occupational Risk Assessment

**Figure 2:**
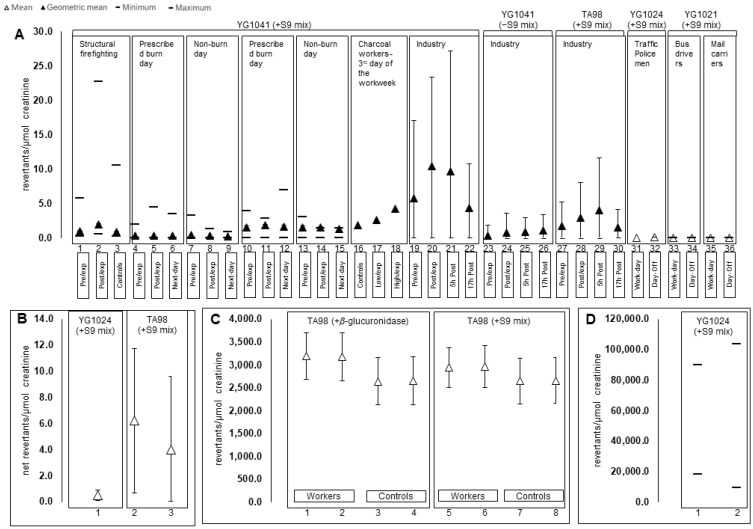
Graphical representations of urinary mutagenicity of the studies that report creatinine adjusted levels as revertants/µmol creatinine (**A**,**C**,**D**), or net revertants/µmol creatinine (**B**). (**B**) coke oven workers; (**C**) textile-industry workers; (**D**) rubber manufacture workers.

Over the last 20 years, the occupational contexts that have been explored include firefighters, coke oven and charcoal workers, industrial workers (textile, chemical, pharmaceutical, rubber manufacturing, truck engine-testing facility), chemical laboratory staff, pharmacy staff, traffic policemen, bus drivers, mail carriers, and farmers (Table 2). Since urine flow has interindividual variance, mutagenicity results should be corrected with creatinine values to ensure comparability between the participants. Figure 2 represents the overall distribution of UM levels that were reported with creatinine urinary concentration normalization. However, this normalization was not performed in some studies [24,31,32,33,34,35].

Firefighters from North America (Canada and the United States of America) were biomonitored during exposure to structural fires and prescribed burns due to the toxicity of woodsmoke. Firefighting was the only occupational context in which the results were expressed in the same units between all the three reported studies (expressed as revertants/µmol creatinine) and the authors also included the same strain (YG1041). The ranges of UM values were 0.29–1.55, 0.28–1.90, 0.26–1.67 revertants/µmol creatinine for pre-exposure, post-exposure, and the morning after exposure to prescribed burns, respectively (Figure 2A, numbers 1–15 from the *x* axis). In the firefighting context, there was a positive correlation between UM and cross-work shift levels of malondialdehyde (a lipid peroxidation biomarker), and 1-hydroxypyrene (a biomarker of PAHs exposure) (Table 2). These findings highlighted a possible association between occupational PAHs exposure, oxidative stress, and the mutagenicity of absorbed chemicals during firefighting activities. Accordingly, recent findings in firefighters have also found relationships between PAHs exposure biomarkers and DNA damage assessed by the comet assay [36,37,38], DNA oxidation assessed by urinary 8-hydroxy-2′-deoxyguanosine levels [39], and lipid peroxidation assessed by 8-isoprostane levels in urine [40]. One of the studies observed a negative correlation between UM and black carbon exposure, and black carbon and PM_2.5_ ratio in firefighters [41]. The authors emphasized that this negative correlation suggests that personal air monitoring is not necessarily correlated with the absorbed dose of firefighters; thus, the health impacts of exposure are best observed via biomonitoring [41].

There was also a weak association between airborne pyrene and benzo(a)pyrene with UM in coke oven workers [35]. The strains TA98 and YG1024 were used for the analysis of UM from Finnish coke oven workers at two separate sampling times (winter and summer) [35]. UM was detected at both sampling times: 328–570 revertants/100 mL of urine for the TA98 strain and 1704–1894 revertants/100 mL of urine for YG1024 strain; the latter levels were higher in workers than in the controls (226–1190 revertants/100 mL of urine) (Table 2). Coke oven workers are also exposed to organic volatile compounds such as PAHs. UM, assessed by TA98, resulted in 6.22 and 4.02 net revertants/µmol creatinine in Asian coke oven workers that had high and low exposure, respectively (Figure 2B, numbers 2 and 3 on the *x* axis). The ratio of the number of induced revertants to the number of revertants in the control sample of urinary extracts from Polish coke oven workers assessed by TA98 and YG1024 were 2.7 and 18.2, respectively (Table 2). On the other hand, Italian coke oven workers presented a UM of 495 revertants/µmol creatinine assayed in YG1024 (Table 2). No correlation with 1-hydroxypyrene was found in Asian coke oven workers [42], yet the European studies [18,35,43,44] found an association between UM and the biomarker of PAHs exposure. However, the Polish study highlighted that UM levels were more related to smoking than the exposure itself [43]. Nevertheless, it is important to understand the mutagenic risk of exposure to a mixture of air pollutants since Kuljukka-Rabb et al., (2002) also found an association between UM and DNA-adduct levels in lymphocytes, which could stimulate carcinogenic pathways [45]. Brazilian charcoal workers who were highly exposed to woodsmoke exhibited an increased UM (YG1041 strain), which was in accordance with their level of exposure, i.e., high > low > no exposure (4.22 > 2.65 > 1.79 revertants/µmol creatinine, respectively; Figure 2A, numbers 16–18 from the *x* axis). In the case of these workers (firefighters, coke oven and charcoal workers), the inhalation route is not the only concern. Dermal absorption of these chemicals through cross-contamination of individual protection equipment and surfaces also needs to be considered [46,47,48,49].

Workers who frequently use organic solvents (e.g., laboratory chemicals), pharmaceutical drugs (e.g., antineoplastic drugs) or who are exposed to chemical factory emissions (e.g., nitrotoluenes, plastic derivatives, rubber manufacturing) can also be at risk of developing health issues [31,32,33,34,50,51,52,53]. UM was assessed in chemical laboratory workers with strains YG1024 (non-detected to 25.1 revertants/mL equivalent of urine) and TA100 (non-detected revertants/mL equivalent of urine). Two strains were used for urinary mutagenic assays in pharmacy workers and pharmaceutical factory workers: TA98 (20.00–84.71% of urinary mutagenic positives and a rate of 7.43–22.43 revertant colonies, respectively) and TA100 (14.71–81.76% of urinary mutagenic positives and a rate of 84.71 revertant colonies, respectively). Workers from a chemical factory that were exposed to dinitrotoluenes and mononitrotoluenes presented 46.2–354.2 revertants/mL equivalent of urine (Table 2). The same strain, YG1041, was used for UM assays in workers from a chemical factory exposed to nitrotoluenes 8 h per day, 5 days a week and compared with factory controls [31]. The controls presented far lower urinary mutagenic levels in urine than factory workers (2.8–8.8 versus 53.7–486.4 revertants/mL equivalent of urine). On the other hand, the urine of workers exposed to plastic monomers presented 79.5% revertant colonies based on a TA98 UM assay (Table 2). In factory workers, UM levels were associated with 2,4,6-trinitrotoluene metabolite urinary concentrations [31], dinitrotoluenes levels in blood and the metabolites concentration in urine [34]. Moreover, a good correlation was observed between air PM_10_ levels and results from the tests performed with TA98 (+S9 mix) (post-shift: 2.89 revertants/µmol creatinine), YG1041(−S9 mix) (pre-: 5.79 and post-shift: 10.42, revertants/µmol creatinine) in workers from a heavy industrial zone (Figure 2A, numbers 19–30 on the *x* axis).

In other industries such textile production workers can be exposed to dyes and other processing chemicals. Only one study assessed the UM of such exposures using TA98 strains and reported a Log mutagenic index of 2940–3190 and 2640–2660 revertants/µmol creatinine in 24 h/urine samples of workers and controls, respectively; no association with smoking habits was found in this study (Figure 2C). In another context, rubber factory workers presented increased UM with 1.82 × 10^7^ revertants/µmol creatinine (YG1024 strain) after a workweek in rubber manufacturing (Table 2). The same strain and the same context were explored in another study in which 24 h-urine samples were collected [53]. Mutagenicity levels ranged between 9049.6 and 103,731.04 revertants/µmol creatinine during the working week versus a range of 18,212.32–89,704.16 revertants/µmol creatinine on a non-workday (Figure 2D). In rubber manufacturing workers, the UM data were correlated with slow acetylation phenotypes, mild skin aberrations and DNA-adducts in the blood [52,53]. Dealing with synthetic chemicals (which can be mutagenic) can result in the accumulation of different effects that can lead to cancer development as well as other diseases [54].

Besides the contribution of industrial and wildland fires to air pollution, urban pollution is also a problem, not only to the general population, but also for occupational workers such as truck engine-testing facility staff, traffic police and professional drivers. Workers exposed to diesel engine exhaust in a testing facility (6.1–107.7 µg/m^3^) presented higher mean UM using YG1041 strains than the controls (13.0 versus 5.6 revertants/mL equivalent of urine; Table 2). There was a positive exposure–response relationship between elemental carbon below the European occupational exposure limit (50 µg/m^3^), which suggests that a safer threshold of exposure may be needed [24]. UM was also performed in samples collected from traffic policemen before the start of a working week and after it ended (after 6 days) [55]. The results showed augmented values after occupational exposure (0.062 versus 0.021 revertants/µmol creatinine) in an assay performed with the YG1024 strain (Figure 2A, numbers 31 and 32 on the *x* axis). The mutagenic activity of the extracts was positively correlated with 1-hydroxypyrene urinary levels in traffic policemen [55]. Another study analyzed urine from bus drivers and mail carriers for mutagenic activity of pollutants to which they were exposed during their working days and days off (Table 2). The UM assay (with the YG1021 strain) revealed no significant difference in the results for working days and days off in bus drivers (2.35 × 10^−6^ versus 2.42 × 10^−6^ revertants/µmol creatinine), whereas mail workers had higher mutagenic levels on working days in comparison with days off (1.25 × 10^−6^ versus 7.6 × 10^−7^ revertants/µmol creatinine) (Figure 2A, numbers 33–36 on the *x* axis). Even though UM did not correlate with 1-hydroxypyrene levels, the authors found that women bus drivers had higher mutagenicity than men drivers, and slow acetylators presented higher levels of mutagenicity than fast acetylators [56]. Due to a slower metabolism, slow acetylators and women may have a prolonged exposure to unmetabolized nitro-PAHs and aromatic amines, which could help to explain the results obtained [56,57]. Lastly, regarding farmers’ exposure to pesticides, André et al. (2002) found that the urine extracts were more mutagenic on at least one *Salmonella* strain (TA97a, TA102, TA100, TA98, with and without metabolic activation) in male farmers who were smokers than in those who were non-smokers (57% versus 96%; Table 2); no association was observed between mutagenicity and the available exposure parameters. However, Lebaily et al. (2003) found a positive correlation between the difference in the mutagenic power of urine in TA102 strain (UM: next-morning after exposure minus morning-day of exposure) and the predicted captan (fungicide) absorbed dose by exposed male farmers (Table 2). Moreover, an association between UM and individual confounders was not observed by any of the studies (Table 2).

The heterogeneity of data collected from the selected studies did not allow a meta-analysis. Most of the published studies to date have clear limitations such as the use of a reduced or variable number of strains, and scarce information on source characterization and the identification of mutagenic organic pollutants in the surrounding environment (air). Moreover, a biomonitoring study needs to account for the interindividual differences related to exposure and health effects. Most studies used a small population sample (*n* < 30), and the UM results were limited by the choice of strains. In some cases, the studied working population was biologically under characterized (i.e., no biomarkers of exposure) or had disproportionate grouping (e.g., smokers versus non-smokers). Nevertheless, the studies highlighted their limitations and acknowledged the need for caution when interpreting their results. The use of a control population with similar demographic characteristics (age, sex, body mass index, smoking habits, metabolism, etc.) is crucial for comparisons. Biomarkers of effect cannot be directly associated with occupational exposure if uniquely characterized. A thorough characterization of exposure with personal air sampling, chemical analysis of metabolites in urine and questionnaires (e.g., exposure, number of career years, lifestyle, etc.) is required when investigating possible health effect associations with the working environment. The fact that occupational specific exposures, such as to PAHs, can have confounders (e.g., consumption of grilled food), highlights the need to identify other exposure sources. The susceptibility of the exposed subjects in relation to their individual metabolic characteristics is also a key factor. Some people are fast metabolizers who excrete absorbed xenobiotics faster than the average, resulting in a weaker health impact.

**Table 2:** Urinary mutagenicity evaluation (revertants/µmol creatinine, presented as geometric mean ± standard deviation (range), unless indicated otherwise) in various occupational exposure contexts over the last 20 years (2001–2021).

Study Population		Occupational Exposure	Urinary Mutagenicity		Correlation with Exposure/Effect (Bio)Markers	Reference
*n*	Age (Mean ± Standard Deviation (Range))		Bacterial Strain	Concentration		
Firefighters		Structural fire combat: 5 consecutive 24-h shifts typically spanning 12 days	*Salmonella typhimurium* YG1041 (+S9 mix) Negative control: DMSO solvent Positive controls: 2-nitrofluorene and 2- aminoanthracene	Pre-fire: 1.01 ± 0.07 (0.19–5.76) Post-fire: 1.90 ± 0.12 (0.51–22.68)	Not explored	[40]
16	34 (25–50)					
Fire station office workers		5 consecutive 24-h shifts typically spanning 12 days (no exposure)		Post-shift: 0.87 ± 0.08 (0.17–10.5)	Not explored	
17	50 (28–62)					
Firefighters		Prescribed burn days and non-burn days (January–July of 2015)	*Salmonella typhimurium* YG1041 (+S9 mix) Negative control: DMSO solvent Positive controls: 2-nitrofluorene and 2- aminoanthracene	Burn day Pre-shift: 0.29 (0–1.98) ^a^ Post-shift: 0.28 (0.01–4.46) ^a^ Next-morning: 0.26 (0–3.46) ^a^ Non-burn day Pre-shift: 0.45 (0–3.24) ^a^ Post-shift: 0.27 (0–1.33) ^a^ Next-morning: 0.25 (0–0.89) ^a^	Positive correlations between cross-work shift (pre- to post) changes in urinary mutagenicity and Malondialdehyde (*p* = 0.0010), 1-hydroxypyrene (*p* = 0.0001).	[58]
12	33 ± 5.4					
Firefighters		Prescribed burn days and non-burn days (2015–2018)	*Salmonella typhimurium* YG1041 (+S9 mix) Negative control: DMSO solvent Positive controls were 2-nitrofluorene in the absence of S9 and 2-aminoanthgracene in the presence of S9.	Burn day Pre-shift: 1.55 ± 0.14 (0.00–3.85) Post-shift: 1.80 ± 0.13 (0.00–2.83) Next-morning: 1.67 ± 0.19 (0.00–6.96) Non-burn day Pre-shift: 1.53 ± 0.19 (0.00–3.08) Post-shift: 1.52 ± 0.13 (0.00–1.36) Next-morning: 1.34 ± 0.13 (0.00–1.38)	Pre-exposure to next-morning change in the mutagenicity was correlated negatively with black carbon exposure and black carbon to PM_2.5_ ratio.	[41]
19	35.0 ± 7.2					
Charcoal workers		After the third day of the workweek in 8 different charcoal companies.	*Salmonella typhimurium* YG1041 (+S9 mix)	Woodsmoke No exposure: 1.79 (1.26–2.54) ^b^ Low exposure: 2.65 (2.01–3.66) ^b^ High exposure: 4.22 (3.27–5.45) ^b^	Difference in wood smoke exposure among nonsmokers was significant (data shown in a graph)	[25]
132	34.05 ± 10.47					
Chemical laboratory staff		Organic solvents	*Salmonella typhimurium* YG1024 and TA100 (+ or − S9 mix) Negative control: DMSO Positive control: 2-anthramine with S9 and sodium azide for TA100 and 4-o-nitrophenyenediamine for YG1024 without S9	TA100 (−S9): 0 ^c^ TA100 (+S9): 0 ^c^ YG1041 (−S9): 0–2.6 ^c^ YG1041 (+S9): 0–25.1 ^c^	Not explored	[33]
29	29.3 ± 7.7					
Pharmacy workers		Occupationally exposed to antineoplastic drugs in Pharmacy Intravenous Admixture Services	*Salmonella typhimurium* strain TA98 and TA100 with or without S9 mix Negative control: DMSO Positive control: 2-nitrofluorene for TA98 and N-methyl-N-nitro-N-nitrosoguanidine for TA100	TA98 (−S9 mix): 15–60 mL urine: 20.00–64.71 ^d^ TA98 (+S9 mix): 15–60 mL urine: 39.41–84.71 ^d^ TA100 (−S9 mix): 15–60 mL urine: 14.71–62.94 ^d^ TA100 (+S9 mix): 15–60 mL urine: 40.00–81.76 ^d^	Mutagenic activity positively correlated with the dose of urine concentrates.	[32]
158	28.23 ± 6.07					
Chemical factory workers		Involved in the production of satchel charges for mining. Exposed to nitrotoluenes (8 h/day, 5 days/week)	*Salmonella typhimurium* YG1041	Mutagenic potency of: Unhydrolyzed urine: 198.8 ± 375.8 ^cc^ Enzymatically hydrolyzed urines: 486.4 ± 535.9 ^cc^ Enzymatically acid-hydrolyzed urines: 53.7 ± 76.5 ^cc^	The levels of urinary metabolites of 2,4,6-trinitrotoluene (determined in enzymatically hydrolyzed urine) correlated best with mutagenicity (*r* = 0.89–0.96, *p* < 0.01; Spearman-rank test).	[31]
78	39.6 ± 8.1					
(Applied to 11 workers and 6 controls)						
Factory controls		Employed in the same factory but were no longer working directly in jobs that would expose them to nitrotoluenes		Mutagenic potency of: Unhydrolyzed urines: 3.9 ± 2.5 ^cc^ Enzymatically hydrolyzed urines: 8.8 ± 7.6 ^cc^ Enzymatically acid-hydrolyzed urines: 2.8 ± 3.2 ^cc^		
25	38.4 ± 99.4					
Coke oven workers		Locksmiths, drivers, loaders, and welder	*Salmonella typhimurium* TA98 and YG1024 (+S9 mix) Negative control: DMSO solvent		Correlated weakly with pyrene and Benzo(a)Pyrene concentrations in breathing zone. Correlated strongly with urinary 1-hydroxypyrene and DNA adduct levels in lymphocytes.	[35]
(March 1994)				TA98: 328 (80–910) ^e^ YG1024: 1894 (370–4850) ^e^		
18	--					
(September 1994)				TA98: 570 (34–1287) ^e^ YG1024: 1704 (56–8240) ^e^		
21	--					
Controls				TA98 (*n* = 6): 226 (88–441) ^e^ YG1024 (*n* = 7): 1190 (750–2096) ^e^		
6–7						
Coke oven workers		High-exposure group	*Salmonella typhimurium* TA98 (+S9 mix) Negative control: DMSO solvent	6.22 ± 5.54 ^f^	No correlation was found between urinary mutagenicity and 1-hydroxypyrene.	[42]
15	42.3 ± 7.8					
Coke oven workers		Low-exposure group		4.02 ± 5.59 ^f^		
22	43.1 ± 10.5					
Coke oven workers		Workers occupationally exposed in a coke oven plant	*Salmonella typhimurium* TA98 (+S9 mix) and YG1024(+S9 mix) Negative control: redistilled water after DMSO extraction (+S9 mix)	Mutagenic rate (TA98): 2.7 (1.5; 4.5) ^g^ Mutagenic rate (YG1024): 18.2 (7.3; 28.4) ^g^	The influence of smoking on urinary mutagenicity was greater than the effect of exposure. Association between urinary mutagenicity and 1-hydroxypyrene.	[43]
50	40.2 ± 7.8					
Coke oven workers		Workers occupationally exposed in a coke oven plant	*Salmonella typhimurium* YG1024 (+S9 mix) Negative control: DMSO Positive control: 2-Aminofluorene	0.495 ± 0.407 ^h^	Urinary mutagenicity was significantly related to occupational PAH exposure given by 1-hydroxypyrene (*r* = 0.41, *p* = 0.0215)	[44]
31	37 (24–53)					
Workers from a heavy industrial zone		Workers in several plants located in a heavy industrialized area in the south of France, potentially exposed to PAHs	*Salmonella typhimurium* TA98 (+S9 mix), YG1041 (+S9 mix) and YG1041 (−S9 mix) Negative control: DMSO solvent	TA98 (+S9 mix) Before work-shift: 1.72 ± 3.54 ^i^ After work-shift: 2.89 ± 5.14 ^i^ 5 h after work-shift: 4.03 ± 7.56 ^i^ 17 h after work-shift: 1.50 ± 2.58 ^i^ YG1041 (+S9 mix) Before work-shift: 5.79 ± 11.31 ^i^ After work-shift: 10.42 ± 12.95 ^i^ 5 h after work-shift: 9.62 ± 17.58 ^i^ 17 h after work-shift: 4.33 ± 6.42 ^i^ YG1041 (-S9 mix) Before work-shift: 0.35 ± 1.51 ^i^ After work-shift: 0.71 ± 2.90 ^i^ 5 h after work-shift: 0.91 ± 1.96 ^i^ 17 h after work-shift: 1.03 ± 2.33 ^i^	A good correlation between air particulate levels and the test results with TA98S (post-shift) and with YG1041 (pre-shift and post-shift). Urinary 1-hydroxypyrene correlated with the test results with YG1041S (pre-shift, after-shift and 5 h after-shift) while 3-hydroxybenzo(a)pyrene correlated with those obtained with YG1041S (after-shift).	[18]
31	37 ± 10					
Truck engine-testing facility staff		Exposure to diesel engine exhaust	*Salmonella typhimurium* YG1041 (+S9 mix)	All: 13.0 ± 10.1 ^cc^ Exposure to 6.1–39.0 µg/m^3^: 6.7 ± 4.8 ^cc^ Exposure to 39.1–54.5 µg/m^3^: 15.15 ± 9.4 ^cc^ Exposure to 54.6–107.7 µg/m^3^: 19.6 ± 12.9 ^cc^	Positive exposure-response trends between elemental carbon and urinary mutagenicity were detected among subjects exposed to elemental carbon concentrations below the European occupational exposure limit (50 µg/m^3^)	[24]
20	43.2 ± 6.5					
Controls		Unexposed		5.6 ± 4.4 ^cc^		
15	39.4 ± 8.8					
Traffic policemen		Working cycle consisted of six consecutive working days followed by two days off.	*Salmonella typhimurium* YG1024 (+S9 mix) Negative control: DMSO Positive control: 2-Aminofluorene	Pre-shift on day 1: 0.021 ± 0.011 ^hh^ Post-shift on day 6: 0.062 ± 0.021 ^hh^	Correlation of 1-hydroxypyrene with mutagenic activity	[55]
58	47 ± 10.2					
Textile industry workers		Dye processing (most commonly used dyes are arylamine-related chemicals)	*Salmonella typhimurium* TA98 +S9 mix or + *β*-glucuronidase (Distinguishes between slow and fast CYP1A2 activity) (24 h-urine sample)	Presence of *β*-glucuronidase: - Slow: 3190 ± 510 ^j^ - Fast: 3180 ± 520 ^j^ Log mutagenic index in the presence of S9 mix: - Slow: 2940 ± 430 ^j^ - Fast: 2960 ± 0.460 ^j^	No association with smoking habits.	[50]
117	29.41 ± 9.71					
Controls				Presence of *β*-glucuronidase: - Slow: 2640 ± 520 ^j^ - Fast: 2650 ± 530 ^j^ Log mutagenic index in the presence of S9 mix: - Slow: 2650 ± 500 ^j^ - Fast: 2660 ± 500 ^j^		
117	27.44 ± 9.27					
Chemical factory workers		Dinitrotoluenes and mononitrotoluenes manufacturing	*Salmonella typhimurium* YG1041 (−S9 mix)	Unhydrolyzed urine: 46.2 ^k^ Enzymatically hydrolyzed urine: 127 ^k^ Acid-hydrolyzed urine: 354.2 ^k^	Urinary mutagenicity correlated with both metabolites of Dinitrotoluenes in urine and Dinitrotoluenes levels in blood. Weak and non-significant correlation with mononitrotoluenes and their metabolites	[34]
24	--					
Plastic factory workers		Vinyl chloride, plastic monomers	*Salmonella typhimurium* TA98 and TA100 strains + *β*-glucuronidase Negative and positive controls commonly accepted for the Ames test were also applied [14].	TA98: 79.46 ^l^	Not explored	[51]
32	--					
Hospital pharmacy staff		Cytostatic drugs		TA98 (− *β*-glucuronidase): 10.99 ^l^ TA98 (+ *β*-glucuronidase): 14.28 ^l^		
10	--					
Pharmaceutical factory workers		Daunomycin and its precursors		TA98 (− *β*-glucuronidase): 22.43 ^l^ TA98 (+ *β*-glucuronidase): 7.43 ^l^ TA100 (− *β*-glucuronidase): 84.71 ^l^		
33	--					
Rubber manufacturing		Workers of nine companies (three rubber tire/belts, five general rubber goods, and one retreading company	*Salmonella typhimurium* YG1024 (+S9 mix) Negative control: DMSO	Non-work day: 18,212.32–89,704.16 ^m^ Work-week 9049.6–103,731.04 ^m^ (24 h urine samples):	Interaction of slow acetylation status on the correlation between urinary mutagenicity and DNA adducts in blood	[53]
104	--					
Rubber manufacturing		Associated with inhalable particulate and dermal exposure	*Salmonella typhimurium* YG1024 (+S9 mix) Negative control: DMSO; Positive control: 2-Aminopyrene	After a workweek: +1.82 × 10^7 n^	Slow acetylation phenotype and mild skin aberrations were associated with an increased urinary mutagenicity.	[52]
105	37.9 ± 9.0					
Bus drivers—work day		Heavily exposed to air pollution	*Salmonella typhimurium* YG1021 (+ S9 mix) Negative control: DMSO Positive control: 2-Aminoanthracene	2.35 × 10^−6^ (−7.0 × 10^−8^– 9.04 × 10^−6^) ^o^	Women bus drivers had higher mutagenic activity than men and slow acetylators had lower mutagenic activity. Exposure to vehicle exhaust increased urinary mutagenic activity and doing exercise in leisure time decreased urinary mutagenic activity. No influence of age, gender, NAT2 phenotype or of lifestyle factors in mail carriers. No correlation was found between individual concentrations of 1-hydroxypyrene and urinary mutagenicity	[56]
57	45 (27–60)					
Bus drivers—day off				2.42 × 10^−6^ (−7.7 × 10^−7^–6.71 × 10^−6^) ^o^		
60	45 (27–60)					
Mail carriers—work day				1.25 × 10^−6^ (−2.74 × 10^−6^–6.72 × 10^−6^) ^o^		
88	38 (20–60)					
Mail carriers—day off				7.6 × 10^−7^ (−7.9 × 10^−7^–2.04 × 10^−6^) ^o^		
5	38 (28–58)					
Farmers		Male farmers spraying chlorothalonil, a fungicide over two spraying seasons (with two farmers participating twice). S0: before the beginning of the working day; S1: the morning of the day of spraying; S2: the evening of the same day after spraying operations S3: in the morning of the day after.	*Salmonella typhimurium* TA97a, TA98, TA100, TA102. (+ and −S9 mix) Different urine concentrations: Negative control: spontaneous revertants were previously established. Positive control: systematically used for each strain in both conditions (not specified).	^p^ TA97a (−S9 mix): non-smokers: S0: n.d.—1.1; S1: 0.8–1.2; S2: 1.0–1.6; S3: 1.0–1.3; smokers: S0:0.9–1.2; S1: 1.0–1.2; S2: 0.9–1.2; S3: n.d.−1.5. TA97a (+S9 mix): non-smokers: S0: n.d.−1.2; S1: 0.9–1.6; S2: 0.9–1.3 S3: 1.0–1.3; smokers: S0: 0.8–1.1; S1: 1.0–1.4: S2: 1.1–1.2; S3: n.d.−1.1. TA98 (−S9 mix): non-smokers: S0: n.d.−1.4; S1: 0.9–1.4; S2: 1.0–1.4; S3: 0.9–1.6; smokers: S0: 0.9–1.2; S1: 1.0–1.1; S2: 1.0–1.3; S3: 1.0–1.3. TA98 (+S9 mix): non-smokers: S0: n.d.—1.3; S1: n.d.−1.3; S2: 1.0–2.1; S3: 1.0–1.5; smokers: S0: 0.9–1.5; S1: 1.1–1.5; S2: 1.2–3.4; S3: 1.3–3.3. TA100 (−S9 mix): non-smokers: S0: n.d.−1.1; S1: 0.9–1.2; S2: 0.8–1.2; S3: 0.9–1.3; smokers: S0: 0.9–1.0; S1: 1.0–1.3; S2: 0.9–1.2; S3: n.d.−1.4. TA100 (+S9 mix): non-smokers: S0: n.d.−1.1; S1: 0.9–1.4; S2: 1.0–1.4; S3: 1.0–2.7; smokers: S0: 0.8–1.1; S1: 1.0–1.4; S2:1.0–1.2; S3: n.d.−1.3. TA102 (−S9 mix): non-smokers: S0: n.d.−1.3; S1: 1.0–1.7; S2: 0.9–1.9; S3: n.d.−1.7; smokers: S0: 1.0–1.2; S1: 1.0–1.2; S2: 1.0–1.2; S3: 1.0–1.4. TA102 (+S9 mix): non-smokers: S0: n.d.−1.2; S1: 0.8–1.2; S2: 0.8–1.2; S3: 0.9–1.3; smokers: S0: 1.0–1.3; S1: 0.8–1.5; S2: 1.0–1.2; S3: 1.0–1.8.	No relationships between the relative changes in the number of revertants (adjusted for urine concentration) and any exposure parameters available: area sprayed, number of tanks prepared and time free of exposure to any pesticide.	[59]
14	36.1 (25–50)					
Farmers		Male fruit growers spraying fungicide captan for apple or pear trees over two spraying seasons (1998 and 2000). S1: morning before the day of spraying. S2: evening of the day of spraying. S3: morning of the day after captan exposure.	*Salmonella typhimurium* TA97a (−S9 mix), TA102 (−S9 mix), YG1041 (+S9 mix) Negative control: DMSO + spontaneous revertants were previously stablished Positive control: Acridine Mutagen ICR 191 (0.4 ng/plate; TA97a), mitomycine C (0.4 ng/plate), and benzo(a)pyrene (0.5 mg/plate) for quality control of S9 mix.	^q^ YG1041 (+S9 mix): S1: 1.29 ± 0.17; S2: 1.42 ± 0.46; S3: 1.34 ± 0.26. TA97a (−S9 mix): S1: 1.04 ± 0.11; S2: 1.09 ± 0.10; S3: 1.07 ± 0.11. TA102 (−S9 mix): S1: 1.11 ± 0.10; S2: 1.08 ± 0.09; S3: 1.07 ± 0.10	For YG1041 results: no association with parameters related to confounding factors, except for smoking consumption effect. TA102: no association with pesticide exposure S1; positive correlation with predicted absorbed dose of captan (*p* < 0.01 linear regression, *p* = 0.03, Spearman’s *r*_o_ = 0.40) at S3 but not at S2; positive correlation (*p* = 0.07 linear regression analysis, Spearman’s *r*_o_ =0.56, *p* = 0.002) was observed between the difference (S3–S1) of the mutagenic power of urine samples (TA102) and the predicted absorbed dose of captan.	[60]
12 (1998)	39 (22–53)					
17 (2000)	40 (20–55)					

DMSO: Dimethyl sulfoxide; NAT2 genotype: grouped into three different phenotypes, including “slow acetylator” (two slow alleles), “intermediate acetylator” (1 slow and 1 rapid allele), and “rapid” acetylator (2 rapid alleles, sometimes referred to as “fast”); PAHs: Polycyclic aromatic hydrocarbons; PM_2.5_: particulate matter of 2.5 microns or less in width; USA: United States of America. ^a^: Data are presented as geometric mean (range); ^b^: Data are presented as geometric mean (95% confidence interval) ^c^: Data are expressed as number of revertants/mL equivalent of urine sample and presented as a range; ^cc^: Data are expressed as number of revertants/mL equivalent of urine sample and presented as mean ± standard deviation; ^d^: Data are expressed as frequency of urinary positive mutagenic and presented as a percentage range; ^e^: Data are expressed as revertants/100 mL of urine and presented as median (range); ^f^: Data are expressed as net revertants/mg creatinine and presented as mean ± standard deviation; ^g^: Data are expressed as ratio of a number of induced revertants to a number of revertants in the control sample and presented as median (95% confidence interval); ^h^: Data are presented as mean ± standard deviation, and converted from net revertants/mmol creatinine to net revertants/µmol creatinine; ^hh^: Data are presented as mean ± standard deviation, and converted from revertants/mmol creatinine to revertants/µmol creatinine ^i^: Data are expressed as revertants/mg creatinine and presented as geometric mean ± standard deviation which was calculated based on the data published by Nikoyan et al. (2007); ^j^: Data are converted from revertants/nmol creatinine to revertants/µmol creatinine and presented as mean ± standard deviation; ^k^: Data are expressed as revertants/mL equivalent of urine and presented as mean; ^l^: Data are expressed as rate of revertant colonies and presented as group mean + 2D; ^m^: Data are expressed as revertants/g creatinine and presented as range (of geometric mean published by Peters et al. (2008)); ^n^: Data are expressed as revertants/g of creatinine and presented as difference between post- and pre-exposure; ^o^: Data are expressed as revertants/mol creatinine and presented as mean (range); ^p^: Data are expressed as range of creatinine-adjusted maximum induction ratio; the ratio of induced vs. spontaneous revertants (induction ratio); calculated from the highest mean of induced revertants (most often obtained with the crude extract) and the mean of spontaneous revertants; ^q^: Data are expressed as the ratio between the highest number of reverse mutations in urine samples and the spontaneous background of the corresponding experiment ± standard deviation.

Despite the observed associations between UM and urinary 1-hydroxypyrene [42,43,44,55,56,58], black carbon exposure and the black carbon to PM_2.5_ ratio [41], dinitrotoluenes levels in blood [34], 2,4,6-Trinitrotoluene metabolite [31], malondialdehyde (biomarker of effect) [58], DNA adducts in blood [35,53], and fungicide exposure [60], UM results should be interpreted with caution. Since the comparison among studies is difficult due to the different strain use, different control selection, and expression of the results (Table 2), more studies, especially cohorts, are warranted.

The evaluation of UM in occupational contexts is yet scarce (*n* = 22, 2001–2021). Nevertheless, the information provided by these preliminary studies is important to comprehend the usefulness of this assay to occupational biomonitoring. Some adjustments are required depending on the specificity of each occupational exposure. For instance, firefighting, charcoal workers, and coke oven workers have similar exposures that involve volatile organic compounds, PM, and other atmospheric pollutants. On the other hand, chemical laboratory/factory and pharmaceutical industry workers could be more vulnerable to other type of substances [61]. Therefore, a well-designed study of workers’ exposures is necessary. Future studies should consider: (i) the best suited bacterial strains (including more than one strain), (ii) the inclusion of both activation and inactivation of metabolic activity of the selected strains and respective controls (negative and positive), (iii) controls for individual confounders (e.g., age, diet, metabolic polymorphisms), (iv) the inclusion of a higher number of analyzed subjects (for increased statistical power), and (v) risk communication (e.g., exposure mitigation). Given the increased development of occupational risk assessment methods, mostly in the human genetics field, UM by Ames test using *Salmonella typhimurium* could be used as a preliminary assay to assess exposure to mutagenic substances in combination with other effect biomarkers (e.g., DNA damage, DNA methylation, microRNA, etc.) to better estimate the potential health impacts related to occupational exposure.

## 6. Conclusions and Future Perspectives

The occupational environment is usually characterized by a rich mixture of xenobiotics that can affect the workers’ health. The Ames assay was developed decades ago as a reliable tool to assess the mutagenicity of several substances and compounds. Urinalysis for mutagenic activity in *Salmonella typhimurium* strains has been used for the evaluation of occupational exposure of firefighters, traffic policemen, bus drivers and mail carriers, and workers from various manufacturing and processing sectors: coke oven, charcoal, textile, chemical, pharmaceutical, rubber and chemical laboratory workers, as well as in farmers. Despite the generated data, the comparison between studies is difficult and, limits the evidence for causality. UM has been correlated with chemical metabolite concentrations in the urine of several types of workers. Still, the different sources of exposure, the complex mixture of substances excreted in urine, the intricacy of the human body and the lack of human models of toxicity for each chemical are significant gaps that prevent its easy implementation as a biomarker of mutagenicity in the occupational biomonitoring context. Future studies should aim to explore these limitations and provide solutions by (i) standardizing the methods for specific occupational exposures; (ii) studying the influence of confounders (e.g., tobacco consumption, age, individual susceptibility, etc.); (iii) characterizing the mutagenic activity of chemicals at the established occupational exposure limit concentrations; and (iv) quantitatively and chemically characterizing urine and performing experimental studies with control exposures at occupational exposure limit levels. In addition, the main stakeholders (regulatory agencies, workers, professional associations, industries, etc.) must be involved to achieve a more integrative interpretation of the obtained results and to explore the potential of the Ames test for the revision of existing occupational exposure limits. This way, UM will be able to provide additional valuable information to stakeholders and assist them in the development and implementation of mitigation strategies for different occupational contexts.

## Figures and Tables

**Table 1:** Positive controls for strains with metabolic activation (**A**) and without metabolic activation (**B**). Adapted from “Guideline for testing of chemicals Test Nº 471: Bacterial Reverse Mutation Test”, 2020 [16].

(A) Strain with Metabolic Activation		(B) Strain without Metabolic Activation	
Chemical	CAS No.	Chemical	CAS No.
9,10-Dimethylantrancene	781-43-1	Sodium Azide	26626-22-8
7,12-Dimethylbenzantrancene	57-97-6	2-Nitrofluorene	607-57-8
Congo Red *	573-58-0	9-Aminoacridine	90-45-9
Benzo(a)pyrene	50-32-8	ICR191	17070-45-0
Cyclophosphamide (monohydrate)	50-18-0 (6055-19-2)	Cumene hydroperoxide	80-15-9
2-Aminoanthracene	613-13-8	Mitomycin C	50-07-7

* For reductive metabolic activation method. Note: 2-Aminoantrancene should not be used as the sole indicator of the efficacy of the S9-mix, which also needs a chemical that requires metabolic activation by microsomal enzymes such as benzo(a)pyrene.

## Data Availability

Not applicable.
